# National Guidelines Not Always Followed When Diagnosing Smear-Negative Pulmonary Tuberculosis in Patients with HIV in Botswana

**DOI:** 10.1371/journal.pone.0088654

**Published:** 2014-02-14

**Authors:** Taurayi A. Tafuma, Rosemary J. Burnett, Diana Huis in 't Veld

**Affiliations:** 1 Department of HIV/AIDS Prevention and Care, Ministry of Health, Gaborone, Botswana; 2 Department of Public Health, University of Limpopo, Medunsa Campus, Pretoria, South Africa; 3 Department of Epidemiology and Social Medicine, University of Antwerp, Antwerp, Belgium; 4 FWO Research Foundation Flanders, Brussels, Belgium; Archivel Farma; Fundació Institut d’Investigació en Ciències de la Salut Germans Trias i Pujol. Universitat Autònoma de Barcelona. CIBERES, Spain

## Abstract

**Background:**

Diagnosis of smear negative pulmonary tuberculosis (SNPTB) is challenging, especially in patients with HIV. The Botswana National Tuberculosis Program (BNTP) guidelines give guidance in diagnosing and treating SNPTB. Patients with chronic cough should be screened for TB by 3 sputum smear investigations. If negative, a chest x-ray (CXR) should be performed. If negative for TB, antimicrobial treatment for other infections should be started. We investigated the clinicians’ use of the guidelines in clinical practice.

**Methods:**

Data regarding the medical history (coughing period), requested and conducted investigations concerning tuberculosis diagnosis (sputum smear or culture or CXR) or alternative diagnoses (sputum microscopy or blood or sputum culture for diagnosis of other organisms), in SNPTB HIV-positive patients (outpatients and admitted patients) from 2006–2009 in a district hospital in Botswana were extracted from all available hospital medical records. Additionally, a survey was done in all doctors diagnosing SNPTB in this hospital using a self-administered questionnaire with questions regarding the application of the BNTP guidelines in practice. Descriptive analyses of collected data were performed to test the compliance to the guidelines.

**Results:**

Data from medical records showed that in 47.0% (132/281) of patients, TB treatment was started without microbiological results from sputum smears. Other methods to rule out or confirm PTB were used in 2.1% (6/281); and 99.6% (280/281) of SNPTB patients had received a CXR. The survey in 7 clinicians found that all always used CXR, and all clinicians requested three sputum results only sometimes. Six out of 7 clinicians started antibiotics before starting TB treatment. Reasons clinicians gave for difficulties in following the guidelines included inability of patients to produce sputum; and laboratory delays in releasing sputum results.

**Conclusion:**

Between 2006 and 2009 a high proportion of SNPTB diagnoses in a district hospital in Botswana were not supported by laboratory investigation.

## Background

Smear-negative pulmonary tuberculosis (SNPTB) is an important clinical and public health problem,[1]–[4] especially in sub-Saharan African countries with high human immunodeficiency virus (HIV) prevalence [4]. Botswana has one of the highest tuberculosis (TB) notification rates in the world and has consistently reported in excess of 590 cases per 100 000 population annually since 2000 [5]. According to the 2007 Botswana National TB Program (BNTP) guidelines which were in use until mid 2012, a patient is suspected of having pulmonary TB (PTB) if he presents with a cough for at least 2 weeks. The guidelines stipulate that clinicians should screen such patients for PTB by requesting sputum smear examinations for acid-fast bacilli (AFB) on three sputum specimens collected over three days [5]. An HIV test should also be performed [5]. If AFB-positive, treatment for TB should be started in all patients. If negative, and an ambulatory patient is HIV-positive, a chest X-ray (CXR) needs to be performed. If TB is unlikely on the CXR, antimicrobial treatment for other infections should be started.

In HIV-negative ambulatory patients antibiotics should be started if the AFB stain is negative. Treatment response should be evaluated before taking a CXR. Those who are seriously ill should be admitted and put on parenteral antibiotics for bacterial infection. If AFB results are negative or pending and there is no improvement on antibiotics after 3–5 days, TB treatment should be commenced [5]. The BNTP guidelines define a SNPTB case as a patient with either: (i) three negative smear results and radiological findings and doctor’s decision to treat for TB; or (ii) negative smear results and a positive culture result for *Mycobacterium tuberculosis* (MTB); or (iii) an inability to produce sputum and highly suspicious radiological and clinical findings which point to PTB [5].

In patients with HIV infection, it is often difficult to confirm the diagnosis of PTB by direct sputum examination. Botswana has a national HIV prevalence of 17.6% [6], thus the frequency of SNPTB is also expected to be high, with one study reporting 22% of PTB cases being SNPTB [17]. Furthermore, in resource-constrained settings, the quality of smear microscopy is often suboptimal due to inadequate sputum collection, incorrect storage, poor staining, reading errors, or poor laboratory services [2]. This leads to clinicians relying mainly on clinical presentation and CXR findings [7]. Ultimately, there are possibilities of over- or under-diagnosis of TB in patients with negative sputum results, resulting in excessive early mortality compared with patients with smear-positive PTB [2], [8]–[9]. In sub-Saharan Africa, TB is often the first manifestation of HIV infection, and is the leading cause of death among HIV-infected patients. SNPTB patients do transmit TB infection to others [10], [11] although to a lesser extent when compared to smear-positive patients.

We investigated the clinicians’ use of the BNTP guidelines, use of other methods to rule out other lung diseases or confirm MTB, and reasons for difficulties in applying the guidelines in practice when diagnosing SNPTB in HIV-positive patients at a district hospital in Botswana. SNPTB management in HIV positive patients had not changed since the 2003 edition of the BNTP guidelines and this study was conducted just prior to Botswana introducing the World Health Organization’s (WHO) 2010 Treatment of Tuberculosis Guidelines [1], and provides valuable baseline information that can be used when evaluating compliance to the WHO guidelines in the future.

## Methods

The study was carried out at a 177 bed district hospital in the southern region of Botswana, which sees approximately 77000 patients annually (outpatients and inpatients). The hospital offers medical, surgical, pediatric, obstetric and gynecological services. There is a radiology department where CXRs are performed, and a microbiology laboratory with expertise in sputum microscopy for AFB, and microscopy, culture and antibiotic sensitivity testing for infections other than MTB. The reference laboratory where MTB culture is performed, is sixty kilometers from the facility. By 2008 the prevalence of HIV in the general population in this district was approximately 16.3% [6]. There were two study populations for this study: (1) records of HIV-positive patients with SNPTB (HIV diagnosis made after a parallel test of whole blood with Uni-Gold™ [Trinity Biotech, Plc] and KHB™ Rapid HIV test kits), and (2) general practitioners who diagnose and treat these patients in the general outpatient department or the medical ward for admitted patients. All available hospital records with complete data of HIV-positive patients, 15 years and above, who completed treatment for SNPTB from 2006–2009 inclusive, were retrieved from the Hospital Records Department and reviewed retrospectively for medical history and investigations requested/conducted. A self-administered questionnaire with closed-ended questions regarding practices in diagnosing SNPTB, was pilot-tested among general practitioners working in clinics in the same district. Written informed consent was obtained from all 8 general practitioners diagnosing and treating SNPTB HIV-positive patients at the hospital before giving them the self-administered questionnaire. Data from records and clinicians were collected on the number of sputum smear results used; period of cough considered; antibiotic trial prior to diagnosis; use of CXR; other methods (blood culture, sputum culture for organisms other than MTB, etc) used to exclude or confirm PTB. Additional data from clinicians were collected on reasons for difficulties encountered when applying the 2007 BNTP guidelines in practice. Data were captured using Microsoft Excel 2003. Epi-Info version 3.4.3 was used for descriptive statistical analyses. This study involved review of patient files. As it was not possible to obtain informed consent from patients, all personal identifiers were delinked from the records. Ethics approval was obtained from both the Medunsa Research Ethics Committee of the University of Limpopo, South Africa, and the Health Research Development Division of the Ministry of Health of Botswana. The district hospital was conveniently selected and the name has not been revealed in this report in order to maintain confidentiality.

## Results

A total of 281 files (128 inpatients and 153 outpatients) were available that fulfilled the inclusion criteria. Seven of the eight clinicians given questionnaires completed them. The eighth clinician went on extended leave out of the country without returning the questionnaire.

### Use of Guidelines

Data on variables stipulated in the guidelines (three sputum results; a cough for at least two weeks; CXR findings; and a trial of antibiotics) were available from the inpatients’ records. There were no data available concerning the response to antibiotics in outpatients’ records. Of the SNPTB inpatients’ and outpatients’ records reviewed, 5.5% (7/128) and 17.6% (27/153) respectively showed that they were started on TB treatment according to BNTP guidelines. Also, 99.6% (280/281) of patients diagnosed with SNPTB had received a CXR, while 47.0% (132/281) were started on TB treatment without sputum results. These results are shown in Table 1.

**Table 1:** Use of Botswana National Tuberculosis Program guidelines to diagnose smear negative Tuberculosis.

	Records review (n = 281)				Clinicians’ self-report (n = 7) n (%)
Guideline		Inpatient(n = 128) n (%)	Outpatient(n = 153) n (%)	Overall(n = 281) n(%)	
Number of smear-negative sputum results *(Guideline* *is three)*	None	76 (59.4%)	56 (36.6%)	132 (47.0%)	*Guideline considered*
					Sometimes: 7 (100%)
					Always: 0 (0%)
	One	20 (15.6%)	30 (19.6%)	50 (17.8%)	
	Two	11 (8.6%)	24 (15.7%)	35 (12.5%)	
	Three	21 (16.4%)	43 (28.1%)	64 (22.8%)	
Period of coughing before TB treatment *(Guideline* *is at least two weeks)*	Not specified	48 (37.5%)	66 (43.1%)	114 (40.6%)	*Guideline considered*
					Sometimes: 3 (42.9%)
					Always: 4 (57.1%)
	<Two weeks	11 (8.6%)	8 (5.2%)	19 (6.8%)	
	≥Two weeks	69 (53.9%)	79 (51.6%)	148 (52.7%)	
Trial of antibiotics	No	29 (22.7%)	*No provision* *on the card*	*Recorded for* *inpatients only*	*Trial considered*
					Sometimes: 1 (14.3%)
					Always: 6 (85.7%)
	Yes	99 (77.3%)	*No provision* *on the card*	*Recorded for* *inpatients only*	
Use of CXR	No	1 (0.8%)	0 (00.0%)	1 (0.4%)	All seven (100%) always request a CXR
					*Diagnosis based on CXR*
					Sometimes: 4 (57.1%)
					Always: 2 (28.6%)
					Never: 1 (14.3%)
	Yes	127 (99.2%)*:*	153 (100%)	280 (99.6%)	
		*Results of CXR*			
		Typical PTB: 115 (41.1%)			
		Other^a^: 155 (55.4%)			
		Normal: 10 (3.6%)			

a Includes “haze features”, “multiple patchy opacities”, “hilar patch infiltrates”, “fluffy infiltrates”, “severe lesions with consolidation”, “bilateral extensive infiltrates”, etc.

### Use of Other Methods

Of the records reviewed 2.1% (6/281) showed that other methods were used to rule out other respiratory conditions as well as to confirm the presence of MTB. These methods are described in Table 2.

**Table 2:** Additional methods used by clinicians to confirm or exclude TB and other infections.

	Records (n = 281)	Response from clinicians (n = 7)		
Methods	Investigation performed n (%)	Never n (%)	Sometimes n (%)	Always n (%)
Blood culture for other infections	3 (1.1)	1 (14.3)	6 (85.7)	0 (0.0)
Sputum microscopy & culture(other organisms)	2 (0.7)	0 (0.0)	5 (71.4)	2 (28.6)
Sputum culture MTB	1 (0.4)	2 (28.6)	5 (71.4)	0 (0.0)

### Reasons for having Difficulties Applying the BNTP Guidelines in Practice

All seven clinicians experienced that some patients with suspected PTB were not able to produce sputum for analysis. Four found that patients did not submit sputum on two consecutive days. Five reported that the long turnover time of the results from the laboratory had an effect on the flow of their management. One did not trust the accuracy of the laboratory results. Three feared losing patients during investigations. Three found the BNTP guidelines difficult to use. Two stated the guidelines were not relevant to clinical practice. All seven reported the absence of the lung disease physician. Four reported that (a) the lack of feedback from the BNTP on the clinicians’ practice contributed to their difficulties with using the guidelines, and (b) feedback from BNTP would make a difference in their clinical practice.

## Discussion

This study showed that only 22.8% of SNPTB cases were confirmed by three negative sputum results, and that clinicians rely mostly on CXR to diagnose TB as nearly half (47.0%) of the patients in this study were started on TB treatment without any sputum results at all. An increase in SNPTB diagnosis in countries with a high burden of HIV has been attributed to HIV co-infection [2], [8], [9]. The occurrence of other HIV-related pulmonary diseases that clinically resemble PTB has been found to increase the false-positive diagnosis of SNPTB in African countries where other diagnostic techniques are unavailable [13].

In Ethiopia it was found that improving adherence to the national diagnostic algorithm increased the accurate detection of both smear-positive PTB and SNPTB in areas with high HIV and TB co-infection rates [13]. Without a standardized clinical work up, rates of misdiagnosis have been estimated to be as high as 35% to 52% [14].

Treatment algorithms sometimes oversimplify the management of patients, especially when patients present with atypical signs and symptoms. Perhaps this is why a few clinicians in this study found the guidelines irrelevant or difficult to use in clinical practice. This difficulty is compounded by the absence of a lung disease specialist, who would have assisted with interpretations of the CXRs as well as atypical clinical presentations. All clinicians reported this as a reason for finding it difficult to use the BNTP guidelines. The sensitivity of sputum microscopy is low especially in patients co-infected with HIV due to a lower rate of caseation necrosis, and consequent lower numbers of AFB in the airway [12] and this could be the reason why one clinician did not trust the accuracy of the laboratory results.

There is a discrepancy between the clinicians’ reported frequency of use of other methods to diagnose other pulmonary diseases or to confirm PTB, and the number of times other methods are actually used according to the records. Although the additional investigations are not mentioned as standard of care in the guidelines, the expectation was that additional investigations which are easily available at the hospital or within reach, would be performed. However, only 2.1% of the records reviewed showed that additional investigations were done. This could be due as well to non-uniform reporting and poor record keeping [15] by clinicians. Alternatively, clinicians may have over-reported the use of other methods. The finding that 47.0% of SNPTB cases had no sputum smear results suggests that sputum is not routinely collected or submitted by patients. Sputum microscopy and culture are very important to rule out other respiratory infections, as was noted in a Ugandan study where patients suspected of PTB were found to have *Pneumocystis (carinii) jiroveci* (38.6%), MTB (24%), pulmonary Kaposi’s sarcoma (11%), pyogenic bacteria (8%) and no obvious cause for the remainder, when their sputa were sent for microscopy and culture [14]. Thus misdiagnosis of PTB can be reduced if appropriate investigations are conducted, particularly in countries with high HIV burdens.

In contrast, all clinicians reported performing CXRs before making a diagnosis of SNPTB, and this was confirmed by the records reviewed. If the records are correct, this could be due to the easy availability of radiological investigations in Botswana [16] leading to more requests for CXRs, and the fact that results are available immediately after the CXR, compared to sputum microscopy and culture which take a much longer time. Although almost half of the clinicians feared losing patients during investigations, the study showed that 59.4% of the reviewed files of inpatients had no sputum results. These patients could have been fully investigated as they would have been kept in the hospital for at least three days for antibiotic treatment.

This study was performed in one district hospital, thus it is not possible to generalise these findings to other clinical practices in Botswana. The patients’ files considered for this study were confined to those who completed TB treatment which may have introduced selection bias. However, the records of those who did not complete TB treatment were excluded due to the difficulties in tracing them, thus this bias was unavoidable. Furthermore, clinicians’ use of the guidelines was self-reported, and although anonymity was ensured to encourage participants to be completely truthful and thus avoid information bias, the discrepancy between the self-reports and patients’ files regarding the use of other methods, points to information bias. Finally, the 2007 BNTP guidelines were replaced mid 2012 by the WHO 2010 Treatment of Tuberculosis Guidelines [1]. However, as there is a limited number of GeneXpert machines, which allow for same-day sputum results, being introduced in the country, there is limited use of the WHO 2010 guidelines and the 2007 BNTP guidelines are still being followed in some districts without access to this relatively new technology.

## Conclusions

A high proportion of SNPTB diagnoses were not supported by any laboratory investigation. Interventions need to be employed to ensure that guidelines are followed when making a diagnosis of SNPTB, as the wrongly labeled SNPTB cases will strain the meagre resources of the BNTP. A larger survey is recommended so that the general trend of the clinicians’ practice throughout Botswana can be evaluated. This study provides valuable baseline information that can be used when evaluating compliance to the WHO 2010 guidelines which are being introduced.
